# Early detection of myocardial dysfunction in a cat that gradually progressed to endomyocardial form of restrictive cardiomyopathy

**DOI:** 10.1186/s12917-021-02987-7

**Published:** 2021-08-14

**Authors:** Takahiro Saito, Ryohei Suzuki, Yunosuke Yuchi, Takahiro Teshima, Hirotaka Matsumoto, Hidekazu Koyama

**Affiliations:** 1 Laboratory of Veterinary Internal Medicine, School of Veterinary Medicine, Faculty of Veterinary Science, Nippon Veterinary and Life Science University, 1-7-1 Kyonan-cho, 180-8602 Musashino-shi, Tokyo, Japan; 2 Sagamihara Animal Medical Center, 10-17-2 Kobuchi, Minami-ku, 252-0344 Sagamihara-shi, Kanagawa, Japan

**Keywords:** Strain, Strain rate, False chordae tendineae, Layer-specific myocardial function, Two-dimensional speckle tracking echocardiography

## Abstract

**Background:**

Restrictive cardiomyopathy (RCM) is a common myocardial disease in cats, characterized by diastolic dysfunction and atrial enlargement without myocardial hypertrophy. Especially, endomyocardial form of RCM, one of the subtypes in RCM, is characterized by endocardial fibrosis, endocardial scar bridging the interventricular septum and left ventricular (LV) free wall, and deformation and distortion of the LV. However, it is unclear how the myocardial dysfunction and the endocardial scar contribute to the pathophysiology of RCM disease progression.

**Case presentation:**

A 3 years and 2 months old, intact male, Domestic shorthaired cat was presented for consultation of cardiac murmur. At the first visit (day 0), the notable abnormal finding was echocardiography-derived chordae tendineae-like structure bridging the interventricular septum and the LV free wall, resulting high-speed blood flow in the left ventricle. Electrocardiography, thoracic radiography and noninvasive blood pressure measurements were normal. No left atrial enlargement was observed, and LV inflow velocity showed an abnormal relaxation pattern. Although there was no abnormality in tissue Doppler imaging-derived myocardial velocity, two-dimensional speckle tracking echocardiography (2D-STE) revealed a decrease in the LV longitudinal strain and an increase in endocardial to epicardial ratio of the LV circumferential strain on day 0. On day 468, obvious left atrium enlargement and smoke like echo in the left atrium were observed. The LV inflow velocity was fused, and the tissue Doppler imaging-derived early-diastolic myocardial velocity of the septal mitral annulus decreased. Regarding 2D-STE, LV circumferential strain was further decreased, and right ventricular strain was additionally decreased. Although the general condition was good, we made a clinical diagnosis of endomyocardial RCM based on the above findings. On day 503, the cat showed the radiographic evidence of pulmonary edema and congestive heart failure signs.

**Conclusions:**

Cats with abnormal LV structure and associated myocardial dysfunction like this case needs careful observation. Additionally, 2D-STE indices may be useful for early detection of myocardial dysfunction in feline RCM.

## Background

Restrictive cardiomyopathy (RCM) is a common myocardial disease in cats, characterized by diastolic dysfunction and atrial enlargement without myocardial hypertrophy [1–4]. Especially, endomyocardial form of RCM, one of the subtypes in RCM, is characterized by endocardial fibrosis, endocardial scar bridging the interventricular septum (IVS) and left ventricular (LV) free wall, and deformation and distortion of the LV [3]. However, it is unclear how the myocardial dysfunction and the endocardial scar contribute to the pathophysiology of RCM disease progression.

Conventional two-dimensional and Doppler echocardiography are used for the clinical diagnosis of feline RCM [1, 2, 5]. Additionally, tissue Doppler imaging (TDI) has been used for the detection of myocardial dysfunction in feline cardiomyopathy [6, 7]. However, TDI measurements are affected by translation of the heart, tethering of surrounding myocardial motions, and Doppler angle dependency [8, 9]. Recently, two-dimensional speckle tracking echocardiography (2D-STE), a new non-invasive method, enables the non-invasive evaluation of the intrinsic myocardial function without angle dependency, and is expected for the early detection and the pathophysiological assessment of the various myocardial diseases in cats [5, 10, 11]. Additionally, 2D-STE enables the layer-specific myocardial function assessment, and we previously described the layer-specific compensation and decompensation of the LV myocardium in cats with RCM and hypertrophic cardiomyopathy [5, 12].

In this report, we describe a cat that did not meet the diagnostic criteria of RCM assessed by conventional echocardiography on the first visit, but had myocardial dysfunction assessed by layer-specific 2D-STE, and report the utility of layer-specific 2D-STE for early detection of feline RCM.

## Case presentation

**Fig. 1:**
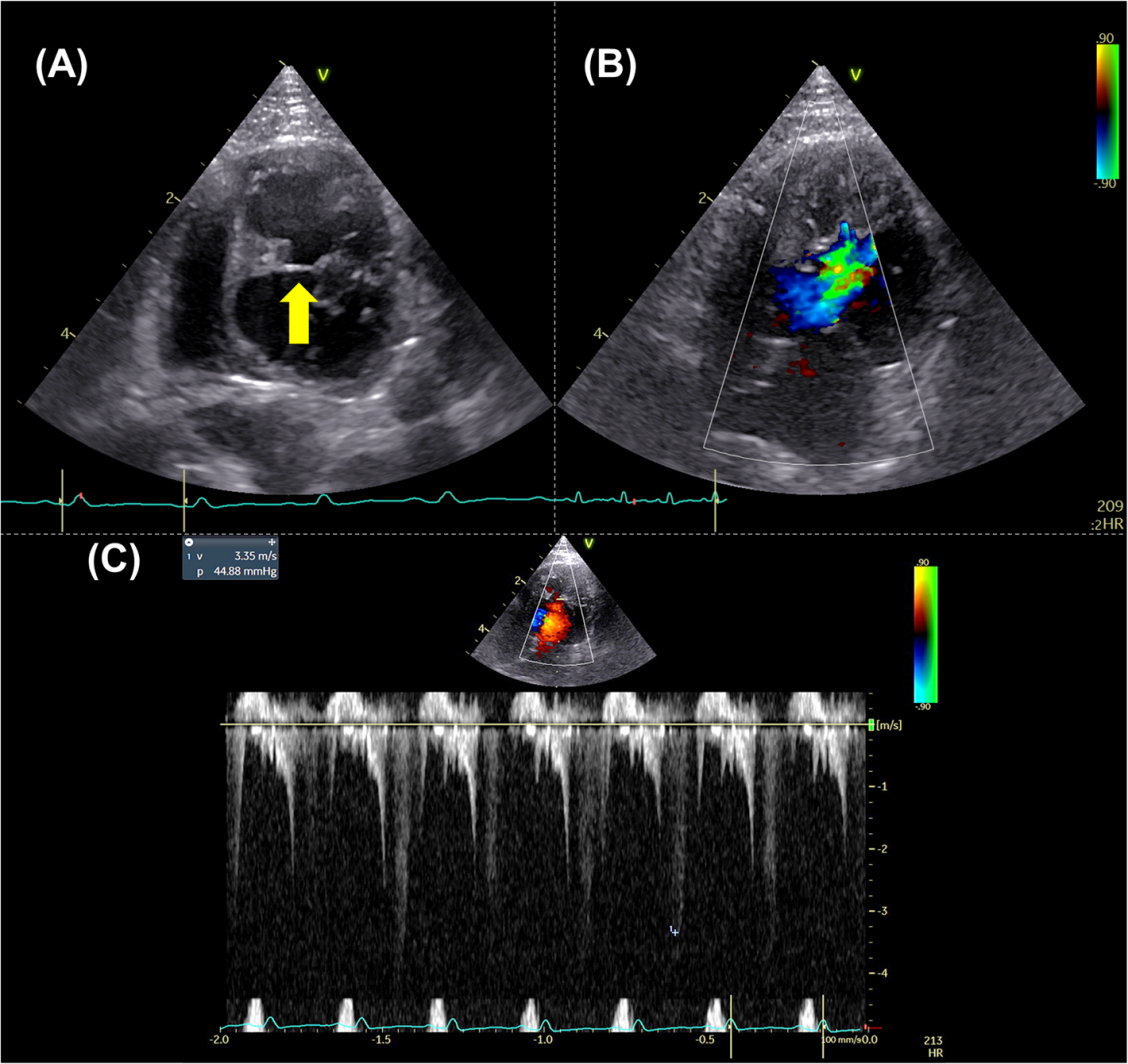
Echocardiography at the first visit (day 0). A false chordae tendineae-like structure bridging the interventricular septum and the left ventricular free wall in the middle left ventricle (arrow) was observed from the left apical four chamber view (**A**). The abnormal structure induced the turbulent midventricular color Doppler flow pattern and spectral Doppler-derived high-speed blood flow in this area (**B, C**)

**Fig. 2:**
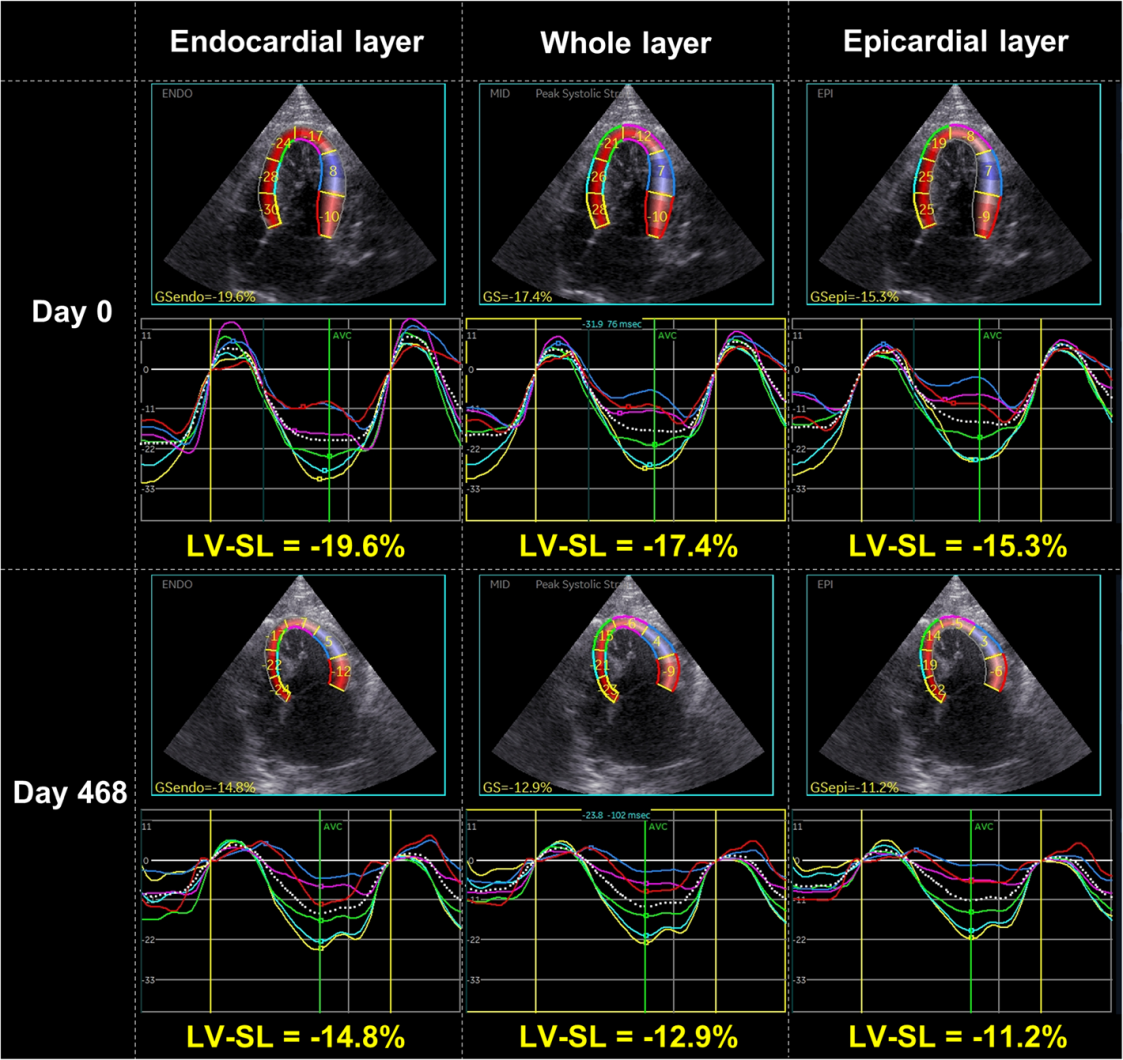
Layer-specific two-dimensional speckle tracking echocardiography-derived left ventricular longitudinal strain (LV-SL) curves on day 0 and day 468. The left curves represent the endocardial layer, the middle represent the whole layer, and the right represent epicardial layer. The whole, endocardial, and epicardial LV-SL on day 0 have already been below the normal range which established from 15 healthy cats, and were further decreased on day 468

**Fig. 3:**
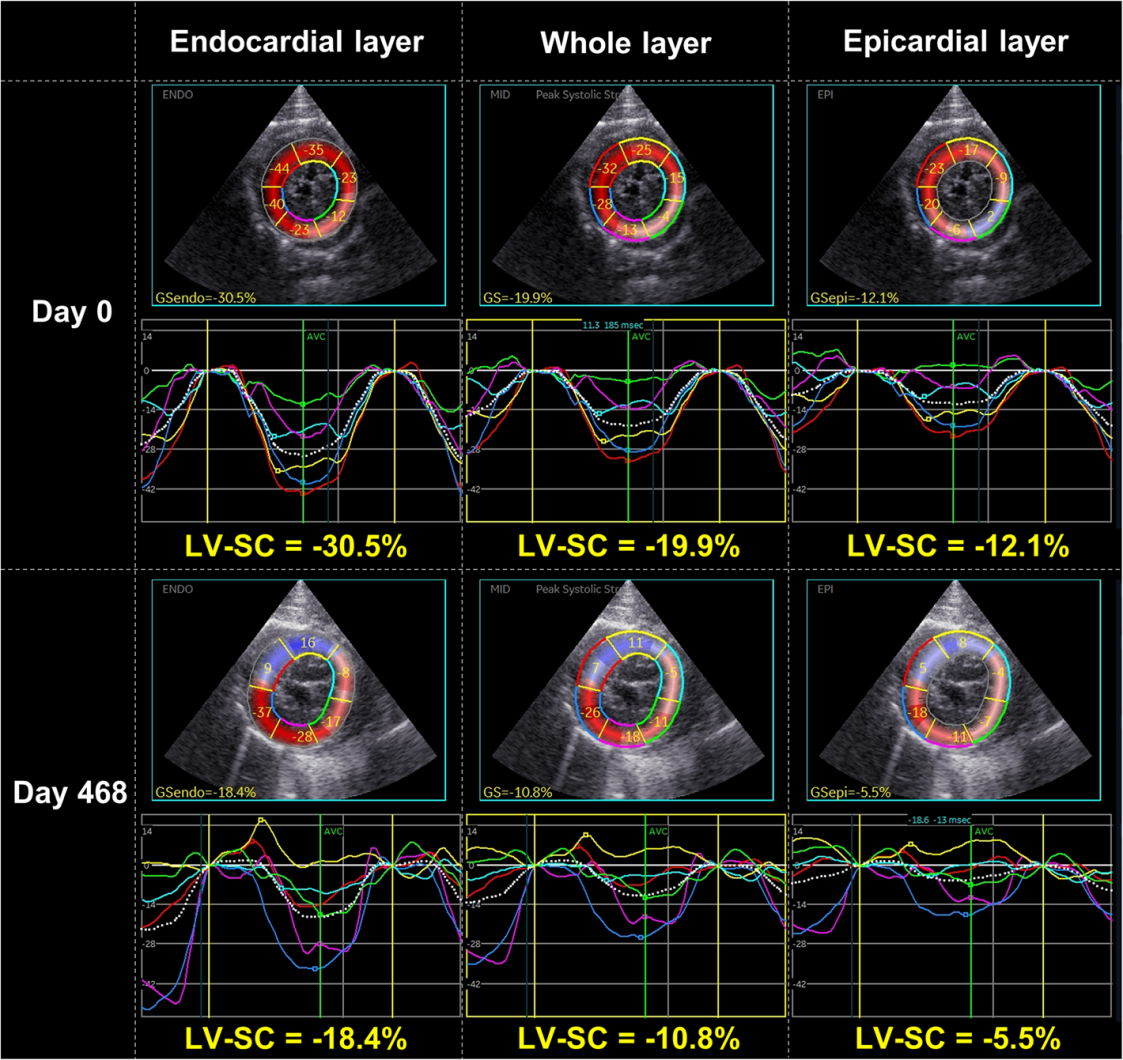
Layer-specific two-dimensional speckle tracking echocardiography-derived left ventricular circumferential strain (LV-SC) curves at the level of papillary muscle on day 0 and day 468. The left curves represent the endocardial layer, the middle represent the whole layer, and the right represent epicardial layer. Only endocardial LV-SC at the level of papillary muscle on day 0 has already been below the normal range which established from 15 healthy cats, and that of the whole and epicardial layers were additionally decreased on day 468

An intact male, domestic shorthaired cat, aged 3 years and 2 months and weighing 4.8 kg, was brought to Nippon Veterinary and Life Science University for a consultation regarding a cardiac murmur. At the first visit (day 0), the cat had no history of relevant clinical signs. On auscultation, a systolic murmur (Levine II/VI) was detected. Electrocardiography, thoracic radiography, and noninvasive blood pressure measurements (systolic blood pressure 125 mmHg) revealed no abnormalities. Conventional two-dimensional and Doppler examinations were performed using a Vivid E95 echocardiographic system (GE Healthcare, Tokyo, Japan), and analyzed using an offline EchoPAC workstation (GE Healthcare, Tokyo, Japan) [5, 11–14]. The non-sedated cat was manually restrained in right and left lateral recumbency. Time-course changes in conventional echocardiographic variables and systolic blood pressure were summarized in Table 1. For comparison, the normal ranges (median [interquartile range]) for all measurements were established from the examinations of 15 healthy cats. Echocardiography detected a chordae tendineae-like structure bridging the IVS and the LV free wall in the middle left ventricle (Fig. 1A). Although there was no hemodynamical abnormality at rest, the structure caused high-speed blood flow (3.4 m/s) in excited conditions (Fig. 1B, C). Early-diastolic and late-diastolic trans-mitral flow velocities (E and A, respectively) showed abnormal relaxation pattern, and no obvious morphological abnormality such as left atrial enlargement was observed. Using 2D-STE, we measured the peak global strain in the longitudinal and circumferential directions (SL and SC, respectively) and the systolic, early-diastolic, and late-diastolic strain rate in the longitudinal and circumferential directions (SrL and SrC, respectively). The SL and SrL were measured at both ventricles. The SC and SrC were measured at the papillary muscle, mitral valve, and apical levels of the left ventricle (PM, MV, and AP, respectively) [13, 15]. Additionally, LV-SL and LV-SC were measured at the whole, endocardial, and epicardial levels for layer-specific myocardial assessment. The mean values of measurements for three consecutive cardiac cycles obtained from high-quality images were used in all analyses. Observer variability of 2D-STE analysis in our laboratory has been described in our previous studies [5, 11–13]. Time-course changes in 2D-STE-derived LV and right ventricular (RV) strain and systolic strain rate were summarized in Tables 2 and 3. At day 0, the LV-SL, early-diastolic LV-SrL, endocardial LV-SC PM, whole and endocardial LV-SC AP, early-diastolic LV-SrC MV, LV-SrC AP, and systolic and early-diastolic RV-SrL were below the 25th percentile of the 15 healthy cats (Figs. 2 and 3).

**Table 1:** Conventional echocardiographic variables at first visit (day 0) and follow-up examinations

	Day 0	Day 89	Day 468	Healthy cats
Echocardiograohic variables				
End-diastolic IVS thickness (mm)	5.9^a^	5.1^a^	5.4^a^	3.8 (3.4, 4.1)
End-diastolic LV free wall thickness (mm)	3.7	3.3^a^	3.3^a^	4.2 (3.6, 4.3)
End-diastolic LV internal dimension (mm)	14.7	17.3^a^	18.5^a^	14.0 (13.5, 15.5)
End-systolic LV internal dimension (mm)	6.8	7.6	11.5^a^	7.2 (6.4, 8.9)
Fractional shortening (%)	53.8^a^	56.1^a^	37.8^a^	49.1 (40.8, 53.1)
LA diameter (mm)	14.3	15.2	19.0	10.9 (9.7, 11.9)
LA to aortic root diameter ratio	1.5^a^	1.5^a^	2.2^a^	1.3 (1.2, 1.4)
E velocity (m/s)	0.46^a^	0.50^a^	0.65 (fused)	0.60 (0.57, 0.73)
A velocity (m/s)	0.71	0.64		0.69 (0.57, 0.73)
E/A	0.6^a^	0.8		0.9 (0.8, 1.2)
S' (cm/s)	4.9^a^	7.7	9.3	8.0 (6.9, 10.5)
E' (cm/s)	11.2^a^	5.8	4.4^a^	7.0 (5.1, 7.6)
Systolic blood pressure (mmHg)	125	135	133	140 (125, 149)

Healthy cat ranges (median [interquartile range]) were established from 15 healthy cats

*A* Late-diastolic trans-mitral flow velocity, *E* Early-diastolic trans-mitral flow velocity, *E’* myocardial velocity of the septal mitral annulus at early-diastole, *IVS* interventricular septum, *LA* left atrium, *LV* left ventricular, *S’* myocardial velocity of the septal mitral annulus at systole

^a^Value that was out of the interquartile range of the 15 healthy cats

**Table. 2:** Layer-specific strain and systolic strain rate assessed by two-dimensional speckle tracking echocardiography at first visit (day 0) and follow-up examinations

	Day 0	Day 89	Day 468	Healthy cats
LV-SL (%)				
Whole layer	-16.2^a^	-13.7^a^	-12.2^a^	-20.8 (-18.3, -22.6)
Endocardium	-18.8^a^	-15.5^a^	-14.7^a^	-23.8 (-21.9, -25.3)
Epicardium	-13.9^a^	-12.2^a^	-10.7^a^	-18.5 (-16.1, -19.8)
Endo/epi	1.4	1.3	1.2	1.4 (1.2, 1.4)
Systolic LV-SrL (s^− 1^)	-3.6^a^	-3.0	-2.5^a^	2.9 (2.3, 3.5)
LV-SC (PM)				
Whole layer (%)	-20.5	-16.3^a^	-11.9^a^	-19.1 (-17.3, -23.9)
Endocardium (%)	-28.5^a^	-27.9^a^	-20.9^a^	-38.7 (-32.7, -48.3)
Epicardium (%)	-15.8	-9.3	-5.8^a^	-7.3 (-6.2, -9.3)
Endo/epi	1.8^a^	3.0^a^	3.6^a^	4.8 (4.1, 6.5)
Systolic LV-SrC (PM) (s^− 1^)	-3.7^a^	-2.8	-2.3^a^	2.8 (2.5, 3.2)
LV-SC (MV)				
Whole layer (%)	-21.0^a^	-28.5^a^	-18.7	-18.1 (-16.5, -20.1)
Endocardium (%)	-35.9	-49.9^a^	-30.9	-31.7 (-25.7, -37.4)
Epicardium (%)	-14.7^a^	-14.0^a^	-10.6	-9.7 (-6.7, -13.5)
Endo/epi	2.4^a^	3.6	2.9	3.1 (2.5, 5.1)
Systolic LV-SrC (MV) (s^− 1^)	-4.2^a^	-4.6^a^	-3.0	-3.1 (-2.7, -3.7)
LV-SC (AP) (%)				
Whole layer (%)	-8.0^a^	-13.0^a^	-5.6^a^	-18.5 (-16.3, -22.4)
Endocardium (%)	-12.0^a^	-20.8^a^	-5.5^a^	-37.2 (-31.9, -42.1)
Epicardium (%)	-6.3	-8.0	-5.9	-6.6 (-5.5, -11.3)
Endo/epi	1.9^a^	2.6^a^	0.9^a^	5.2 (3.8, 6.2)
Systolic LV-SrC (AP) (s^− 1^)	-2.2^a^	-2.8	-1.6^a^	-2.9 (-2.7, -3.2)
RV-SL (%)				
Whole layer (%)	-31.7	-32.5	-27.2^a^	-32.9 (-28.2, -40.2)
Endocardium (%)	-35.7	-35.7	-33.3	-35.8 (-29.9, -42.3)
Epicardium (%)	-28.2	-29.9	-22.4^a^	-30.7 (-26.1, -38.3)
Endo/epi	1.3	1.2	1.5^a^	1.3 (1.2, 1.3)
Systolic RV-SrL (s^− 1^)	-7.1^a^	-7.4^a^	-8.0^a^	-5.8 (-4.8, -6.9)

Healthy cat ranges (median [interquartile range]) were established from 15 healthy cats

*AP* apical level, *LV* left ventricular, *MV* mitral valve level, *PM* papillary muscle level, *RV* right ventricular, *SC* circumferential strain, *SL* longitudinal strain, *SrC* circumferential strain rate, *SrL* longitudinal strain rate

^a^Value that was out of the interquartile range of the 15 healthy cats

**Table. 3:** Diastolic strain rate assessed by two-dimensional speckle tracking echocardiography at first visit (day 0) and follow-up examinations

	Day 0	Day 89	Day 468	Healthy cats
LV-SrL (s^− 1^)				
Early-diastole	3.3^a^	2.2^a^	1.6^a^	5.2 (4.1, 5.8)
Late-diastole	5.4^a^	3.1	2.9	2.3 (1.5, 4.1)
LV-SrC (PM) (s^− 1^)				
Early-diastole	3.7	2.8^a^	2.3^a^	3.4 (3.0, 4.0)
Late-diastole	2.7	2.7	2.2	2.7 (1.7, 3.4)
LV-SrC (MV) (s^− 1^)				
Early-diastole	2.6^a^	4.9	3.4	3.2 (2.7, 3.6)
Late-diastole	3.0	3.9^a^	3.4^a^	2.5 (1.8, 3.2)
LV-SrC (AP) (s^− 1^)				
Early-diastole	0.9^a^	0.4^a^	0.3^a^	3.5 (2.4, 4.5)
Late-diastole	1.3^a^	3.1	1.5^a^	3.1 (2.4, 4.5)
RV-SrL (s^− 1^)				
Early-diastole	2.9^a^	4.1^a^	2.4^a^	5.6 (4.8, 6.7)
Late-diastole	6.1	3.5^a^	4.9	4.6 (3.8, 6.6)

Healthy cat ranges (median [interquartile range]) were established from 15 healthy cats

*AP *apical level, *LV* left ventricular, *MV* mitral valve level, *PM* papillary muscle level, *RV *right ventricular, *SC* circumferential strain, *SL* longitudinal strain, *SrC* circumferential strain rate, *SrL* longitudinal strain rate

^a^Value that was out of the interquartile range of the 15 healthy cats

On day 89, a decrease was noted in LV-SL and LV-SC at PM, and the early-diastolic LV-SrC PM was found to be below the 25th percentile of the 15 healthy cats. However, since no significant changes were observed in the general condition and echocardiography, no treatment was started.

On day 468, although there were no clinical signs, substantial left atrial (LA) enlargement based on LA to aortic root diameter ratio and smoke-like echo in the LA were observed. The end-diastolic LV internal dimension had increased. We also noticed an increase in heart rate (238 bpm). Although the high-speed blood flow in the left ventricle had increased to 4.3 m/s, fused E and A (0.65 m/s), and diastolic dysfunction based on early-diastolic myocardial velocity of the septal mitral annulus (E’) were observed. Regarding 2D-STE, LV-SC and LV-SrC AP were severely decreased, and the epicardial LV-SC PM, systolic LV-SrC PM, and whole and epicardial RV-SL were additionally below the 25th percentile of the 15 healthy cats (Tables 2 and 3; Fig. 3). We arrived a clinical diagnosis of endomyocardial form of restrictive cardiomyopathy (RCM) based on these findings and started oral administration of β-blockers (carvedilol, DAIICHI SANKYO COMPANY, 0.12 mg/kg, BID) and clopidogrel (DAIICHI SANKYO COMPANY, 18.75 mg/head, SID) to improve diastolic function and prevent thrombosis [2, 5]. The dose of carvedilol was increased to 0.24 mg/kg 2 weeks after starting medications.

On day 503, the cat visited the referral hospital with a complaint of dyspnea, and was diagnosed with pulmonary edema and congestive heart failure. After emergency treatment with oxygen and furosemide, we additionally administered oral pimobendan (Kyoritsu Seiyaku Corporation 0.3 mg/kg, BID). On day 523, the general condition of the cat was good.

## Discussion and conclusions

We reported the early detection of myocardial dysfunction by 2D-STE which conventional echocardiography could not detect on day 0. In present, clinical diagnosis of RCM is performed according to the echocardiographic evidence of atrial enlargement and diastolic dysfunction, using LA to aortic root diameter ratio, trans-mitral flow pattern, and TDI. However, trans-mitral flow is often fused due to tachycardia, and angle dependency and loading condition would affect the TDI-derived assessment of the myocardial function. Therefore, conventional echocardiography, including TDI, may be insufficient for the clinical diagnosis of RCM.

In this case, we assessed myocardial function using 2D-STE, a newly developed echocardiographic tool to evaluate intrinsic myocardial function. Layer-specific myocardial assessment has been demonstrated to be useful for the early detection of myocardial dysfunction in cats with asymptomatic hypertrophic cardiomyopathy, before the occurrence of LV hypertrophy [14]. At day 0, 2D-STE revealed a decrease in whole LV-SL and whole LV-SC AP. A decrease in LV-SL has been reported to be associated with myocardial fibrosis [16]. Additionally, early-diastolic LV-SrL, early-diastolic LV-SrC MV and AP, and early-diastolic RV-SrL were below the normal range. These impaired indices may have sensitively reflected myocardial dysfunction due to fibrosis in RCM, which could not be detected by conventional echocardiography. On day 89, the LV-SL, early diastolic LV-SrL, and early-diastolic LV-SrC PM and AP were further decreased and the whole LV-SC PM and early-diastolic LV-SrC PM were below the normal range. On day 468, the systolic LV-SrC PM and whole RV-SL were additionally below the normal range. These findings suggest that widespread myocardial dysfunction in the ventricles is associated with the onset of congestive heart failure (seen on day 503). Thus, 2D-STE-derived assessment of myocardial function may be useful in the early detection of the myocardial dysfunction and the disease progression of RCM.

Layer-specific 2D-STE revealed that endocardial LV-SC PM, and endo/epi in the LV-SC PM and AP were decreased at day 0, which has been reported previously in cats with endomyocardial form of RCM [5]. It has been reported that, in hypertrophic cardiomyopathy, endocardial lesions are prominent, and that endocardial function is enhanced by compensation for the decreased epicardial function [12]. Conversely, in this case, the endocardial circumferential contractility may be potentially reduced due to myocardial fibrosis, and the maintained epicardial function might reflect compensation for impaired endocardial function, resulting the decrease in endo/epi in the LV-SC PM and AP. These myocardial functional abnormalities may provide additional tools to distinguish the type of feline cardiomyopathy.

Although LV-SC MV was not impaired, LV-SC PM and AP were below the normal range from day 0. The abnormal LV structure and associated abnormally high-speed blood flow in the middle of the left ventricle due to the abnormal chorda tendineae-like structure may have contributed to further apical myocardial damage through increase in wall stress and myocardial ischemia due to afterload. Therefore, in cats with an abnormal chorda tendinea-like structure in addition to myocardial dysfunction like this case, RCM might progress rapidly and result in congestive heart failure.

There were several limitations in this case. First, there is no histopathological findings to make a definitive diagnosis and assess myocardial histopathological alterations. Second, although no medication was given on day 0, the some medications, such as β-blockers and pimobendan, might affect the echocardiographic variables including 2D-STE indices.

In conclusion, abnormal LV structure and associated myocardial dysfunction may contribute to the pathogenesis of RCM, disease progression, and heart failure. Cats with these findings, even without LA enlargement, may require careful follow-up. Further research including pathological examination is desired by accumulating the number of cases in the future.

## Data Availability

The datasets used and/or analyzed during the current study are available from the corresponding author on reasonable request.

## Abbreviations

2D-STE: Two-dimensional speckle tracking echocardiography
A: Late-diastolic transmitral flow velocitiy
AP: Apical level
E: Early-diastolic transmitral flow velocitiy
E': Early-diastolic myocardial velocity of the septal mitral annulus
LA: Left atrial/left atrium
LV: Left ventricle/left ventricular
MV: Mitral valve level
PM: Papillary muscle level
RV: Right ventricle/right ventricular
SC: Circumferential strain
SL: Longitudinal strain
SrC: Circumferential strain rate
SrL: Longitudinal strain rate
TDI: Tissue Doppler imaging
